# Losses along the tuberculosis sputum sample referral cascade for Mpongwe District, Zambia

**DOI:** 10.4102/phcfm.v15i1.3710

**Published:** 2023-02-21

**Authors:** Lyson Nkhoma, Josphat Bwembya, Edwin Chansa, Ramya Kumar, Ibou Thior, Victoria Musonda, Gershom Chongwe, Alwyn Mwinga

**Affiliations:** 1 Mpongwe District Health Office, Ministry of Health, Mpongwe, Zambia; 2 United States Agency for International Development Eradicate TB Project, PATH, Lusaka, Zambia; 3 Research Directorate, Zambart, Lusaka, Zambia; 4 Department of HIV, TB and Viral Hepatitis, PATH, Washington DC, United States; 5 Department of Research, Tropical Diseases Research Centre, Ndola, Zambia

**Keywords:** tuberculosis, sputum, sample, referral, cascade, losses, examination, diagnosis

## Abstract

**Background:**

In resource limited-settings, timely tuberculosis (TB) diagnosis depends upon referral of sputum samples from non-diagnostic to diagnostic facilities for examination. The TB programme data for 2018 suggested losses in Mpongwe District’s sputum referral cascade.

**Aim:**

This study aimed to identify the referral cascade stage where loss of sputum specimen occurred.

**Setting:**

Primary health care facilities in Mpongwe District, Copperbelt Province, Zambia.

**Methods:**

Data were retrospectively collected from one central laboratory and six referring health facilities between January and June 2019, using a paper-based tracking sheet. Descriptive statistics were generated in SPSS version 22.

**Results:**

Of the 328 presumptive pulmonary TB patients found in presumptive TB registers at referring facilities, 311 (94.8%) submitted sputum samples and were referred to the diagnostic facilities. Of these, 290 (93.2%) were received at the laboratory, and 275 (94.8%) were examined. The remaining 15 (5.2%) were rejected for reasons such as ‘insufficient sample’. Results for all examined samples were sent back and received at referring facilities. Referral cascade completion rate was 88.4%. Median turnaround time was six days (IQR = 1.8).

**Conclusion:**

Losses in the sputum referral cascade for Mpongwe District mainly occurred between dispatch of sputum samples and receipt at diagnostic facility. Mpongwe District Health Office needs to establish a system to monitor and evaluate the movement of sputum samples along the referral cascade to minimize losses and ensure timely TB diagnosis.

**Contribution:**

This study has highlighted, at primary health care level for resource limited settings, the stage in the sputum sample referral cascade where losses mainly occur.

## Background

Globally, tuberculosis (TB) is one of the top 10 causes of death and the leading cause from a single infectious agent (besides human immunodeficiency virus and aquired immunodeficiency syndrome [HIV/AIDS]).^1^ In 2019, TB caused an estimated 1.4 million deaths worldwide.^1^ People living in low- and middle-income countries, especially those in sub-Saharan Africa, are at an increased risk of developing and dying from TB because of the increased susceptibility caused by the high prevalence of human immunodeficiency virus (HIV) infection.

Low case detection is one of the biggest challenges to TB control efforts globally and Zambia in particular. In 2018, the World Health Organization (WHO) estimated that there were 62 000 new cases of TB in Zambia. Of these, only 35 071 TB patients were detected and started on TB treatment^2^ – meaning that as many as 25 000 individuals with active TB disease were not detected and were at risk of continuing to transmit the disease in their communities. A major factor contributing to under-diagnosis of TB in resource-limited settings is the limited number of health facilities with TB diagnostic capabilities.^3^ As a result of this, sputum specimens from health facilities without diagnostic equipment must be referred for examination at a zonal or central testing laboratory. But because of long distances to testing laboratories, poor road infrastructure, and weak tracking systems, among many other factors, referred specimens get lost along the referral cascade.^4,5,6^

In the context of TB programming, a cascade is said to be a model for evaluating patient retention across sequential stages of care required to achieve a successful treatment outcome.^7^ Each cascade stage constitutes a step and a gap. The step represents the absolute number of individuals achieving a point in care, while the gap represents the difference between steps, or individuals with suboptimal care or outcomes. In this way, a cascade helps to easily quantify gaps in care delivery and identify areas in which quality of care could be improved.^7^

In the same manner, a sputum referral cascade can be said to be a sequence of stages required to ensure successful completion of the referral cycle. A study conducted by the Zambian Ministry of Health, with support from the Japan International Cooperation Agency, characterized a sputum referral cascade as consisting of seven stages (Table 1).^8^ Delays and inefficiencies at any of these stages can lead to loss of sputum samples, which in turn lead to delayed TB diagnosis and treatment initiation.

**TABLE 1:** Stages in an ideal sputum sample referral cascade.

Stage	Activity	Responsibility
Stage 1	Sputum collection from presumptive client, and registration in TB presumptive register	Referring health facility
Stage 2	Request for sample pick-up	Referring health facility
Stage 3	Sample transportation	Referring health facility
Stage 4	Sample arrival at laboratory	Referral facility
Stage 5	Laboratory tests and records in the TB register	Referral facility
Stage 6	Result returned	Referral facility
Stage 7	Result received and recorded in the presumptive TB register	Referring health facility

*Source:* Ministry of Health. Study on TB sputum sample referral system in Choma and Kalomo Districts, Southern Province, Zambia. Lusaka: Ministry of Health; 2019.

TB, tuberculosis.

In a prospective study conducted in South Africa, 11% of sputum samples and results were lost along the referral cascade. Some of these losses were attributable to leakage of sputum during transportation.^9^ Several programmatic factors that are related to sample handling and transportation, such as long distances to testing laboratories, have also been blamed for losses in the sputum referral cascade.^10^ In China, a study found losses at almost every stage of the referral cascade. A lack of a sample tracking system was seen as a major cause of losses of sputum samples in this study.^11^ These findings were similar to results reported in South Africa^9^ and Zimbabwe.^5^

To ensure optimum performance of the sputum referral system, the Global Laboratory Initiative recommends for the availability of: (1) management teams, including focal point persons at referring health facilities, (2) adequate capacity at testing laboratory, (3) a reliable transport system and scheduling of referral pathways, (4) recording and reporting tools, (5) standard operating procedures (SOPs), (6) training and sensitisation of staff at all the stages of the referral cascade, (7) a communication system that notifies a receiving laboratory of an incoming shipment (specimens) and the referring site that the shipment was received at the laboratory and (8) regular monitoring and evaluation of system performance at each stage of the referral cascades.^6^

The Mpongwe District Health Office in the Copperbelt Province of Zambia has been operating a motorbike sputum specimen courier system since 2018. As a result of limited resources, this courier system is limited to 6 out of 22 non-diagnostic centres (i.e. health facilities without TB testing equipment). The 2018 records at the six non-diagnostic facilities showed that 266 presumptive TB patients submitted sputum samples that needed to be referred for examination at diagnostic centres (i.e. health facilities with TB testing equipment). Records at diagnostic centres, however, showed that only 192 (72%) of these samples were received.^12^ This represented a 28% gap between the number of samples submitted by patients and how many reached the diagnostic facilities, an indication that sputum samples were being lost along the referral cascade. It was, however, not clear at which stage these losses were occurring. This study was conducted to review the motorbike courier system operated by Mpongwe District Health Office by: (1) analysing the sputum specimen referral cascade for the period January to June 2019 as a way of identifying points of losses; (2) determining the turnaround times (TATs) from specimen submission by patient to the delivery of results to the referring centre; and (3) assessing for availability of systems and tools needed to operate a sputum courier system. The study was conducted as part of the operational research capacity building programme for the United States Agency for International Development (USAID) Eradicate TB Project.

## Research methods and design

### Study design

This was a cross-sectional study. We retrospectively tracked the movement of sputum samples for presumptive TB patients from referring facilities to the testing laboratory and test results from the testing laboratory to referring facilities. We also assessed the availability of systems and tools necessary for managing the sputum courier system in Mpongwe District.

### Setting

This research was conducted in Mpongwe District, located in the rural part of Copperbelt Province of Zambia, about 48 km away from the mining town of Luanshya. The official population of Mpongwe District is currently estimated to be 123 613.^12^ The district has 25 health facilities, of which 3 are TB diagnostic centres and 22 are non-diagnostic centres. Two of the three diagnostic centres have both GeneXpert and microscopy platforms, while one only provides microscopy services.

Six out of the 22 non-diagnostic centres are serviced by a motorbike courier system operated by the District Health Office. In this system, a motorbike rider goes round the six non-diagnostic centres to pick up sputum samples and delivers them to diagnostic centres for testing. The same courier system (motorbike) is used to transport test results from diagnostic centres back to the non-diagnostic centres. Patients presenting at the six non-diagnostic centres with symptoms suggestive of TB are asked to provide a sputum sample. Their personal details (name, age and sex) are recorded in the presumptive TB register. A laboratory request form (which accompanies the samples) is prepared by the referring facility and then the samples are prepared for referral. The presumptive TB register also has a provision for recording the date the sample was referred and the date results were received at the referring facility. At the diagnostic facility, received samples are logged into the sample reception book. This book indicates the date that the sample was received and whether or not the sample was moved to the bench for processing. The sample receipt book also maintains the patient’s unique identifiers. Test results are recorded in the laboratory TB register. The laboratory TB register keeps records of when the sample was processed, the results, and maintains the patient’s unique identifiers. The sample request form is updated by the laboratory staff member at the diagnostic facility with results and the date of processing the sample. This laboratory form is sent back to the referring facility through the same motorbike courier service. Upon receipt of the laboratory form at the referring facility, the results section in the specimen submission book and the presumptive TB register are updated, and the laboratory form is filed in the patient record.

Patients presenting with symptoms suggestive of TB at the 16 non-diagnostic centres that are not serviced by the motorbike courier system are referred to TB diagnostic centres for further investigations. In some instances, healthscare workers collect sputum samples from patients and refer them to diagnostic centres using any means of transport available to them.

### Study population and sampling strategy

The study population comprised all the six non-diagnostic facilities in Mpongwe District that were serviced by the motorbike sputum specimen courier system. We used a total population sampling technique to include in our study all the six facilities that were participating in the motorbike sputum courier system that the District Health Office operated. Sputum samples for all presumptive TB patients that presented at the six non-diagnostic sites between 01 January and 30 June 2019 were retrospectively tracked to establish proportions that successfully passed through the various stages of the sputum referral cascade. The period from January to June 2019 represented the most recent cohort of presumptive TB patients at the time this study was conceptualisation (i.e. November 2019).

### Data collection

Data for this study were collected by trained members of the operational research team. The study collected secondary data using two separate paper-based checklists. The first checklist was used to retrospectively track sputum samples through the various stages of the sputum referral cascade. The data collection process began by collecting identifiers (i.e. name, age, sex, address and file number) of presumptive TB patients who presented at the six non-diagnostic sites between 1 January and 30 June 2019 from the presumptive TB register. These identifiers were used to track samples from one register to another. Data on sputum sample submission and sample referral status were also collected from the presumptive register at the non-diagnostic facility. The name of the facility that the sample was referred to was obtained from the laboratory sample request form, whereas the sample receipt book at the diagnostic facility provided data for samples that reached the diagnostic facility. Sputum examination status and results were collected from the laboratory TB register at the diagnostic facility. Data on results sent by the diagnostic facility, including the date of dispatch, were collected from the laboratory TB registers. Data on results received at non-diagnostic facilities, including date of receipt, were collected from the updated results section in the presumptive TB registers.

The second checklist was used to collect data on the availability of systems and tools needed to operate a sputum courier system. This included trained staff, transport, recording and reporting tools, SOPs, and communication and monitoring systems.

### Data analysis

Data from paper-based extraction tools were double entered into an electronic database made in SPSS Version 22, where it was checked for errors and completeness and later analysed. Descriptive statistics such as frequencies and percentages of sputum samples at every stage of the referral cascade were summarized using frequency tables. The following proportions were calculated: (1) proportion of presumptive TB patients who submitted sputum samples at non-diagnostic sites, (2) proportion of sputum samples that were referred to diagnostic centres for testing out of those that were submitted to non-diagnostic sites, (3) proportion of sputum samples that were received at diagnostic centres out of those referred by non-diagnostic sites, (4) proportion of sputum samples that were examined out of those received at diagnostic centres, (5) proportion of sputum samples whose results were sent to non-diagnostic sites out of those examined at diagnostic centres, (6) proportion of sputum samples whose results were received at non-diagnostic sites out of those sent by diagnostic centres and (7) proportion of sputum samples whose results were received at non-diagnostic sites out of those submitted by patients (i.e. referral cycle completion rate).

The average sputum sample TAT was calculated by subtracting the date the sputum sample was submitted by the patient at the non-diagnostic centre from the date the laboratory results were received at the diagnostic centre. Percentages were also calculated for facilities regarding their systems readiness to operate a sputum courier system such as trained staff, transport, recording and reporting tools, SOPs, and communication and monitoring systems.

### Ethical considerations

Ethical approval for this study was sought from the University of Zambia Biomedical Research Ethics Committee (UNZABREC) (No. 805-2020). Authority to conduct research was also obtained from the Ministry of Health through the National Health Research Authority (NRHA). The study was determined to be a non-human subjects research. Copies of authorisation letters obtained from the institutions mentioned above were given to the District Health Director (DHD) for Mpongwe and Health Centre in charge as a way of obtaining their clearance to collect data. As this study utilized secondary data, no informed consent forms were administered.

## Results

### Baseline characteristics

A total of 328 people with presumptive pulmonary TB were included in this study. The median age was 39 years (IQR: 27, 51). There were 181 (55.2%) males and 147 (44.8%) females. Of these, 116 (35.3%) were HIV positive, 140 (42.7%) were HIV negative, and 72 (22.0%) had unknown HIV status. The median distance from referring health facility to the diagnostic facility was 23 km (IQR: 23, 80) (Table 2).

**TABLE 2:** Characteristics of people with presumptive pulmonary tuberculosis in Mpongwe District, Zambia (January–June 2019), *N* = 328.

Variable	Frequency	Percentage
**Age in years**		
Below 15	22	6.7
15–29	71	21.6
30–44	109	33.2
45–59	77	23.5
60 and above	47	14.3
Missing	2	0.6
Median (IQR)	39 (27, 51)	
**Sex**		
Male	181	55.2
Female	147	44.8
**HIV status**		
Negative	140	42.7
Positive	116	35.3
Unknown	72	22.0
**Distance to diagnostic site in kilometres**		
15–50	200	61.0
51–85	111	33.8
86–120	17	5.2
Median (IQR)	23 (23, 80)	
**Sample referred from facility with TB focal point person**		
No	147	44.8
Yes	181	55.2
**Sample referred from facility with refrigerator**		
No	177	54.0
Yes	151	46.0

IQR, interquartile range; HIV, human immunodeficiency virus; TB, tuberculosis.

### Sputum referral cascade for Mpongwe District

**FIGURE 1:**
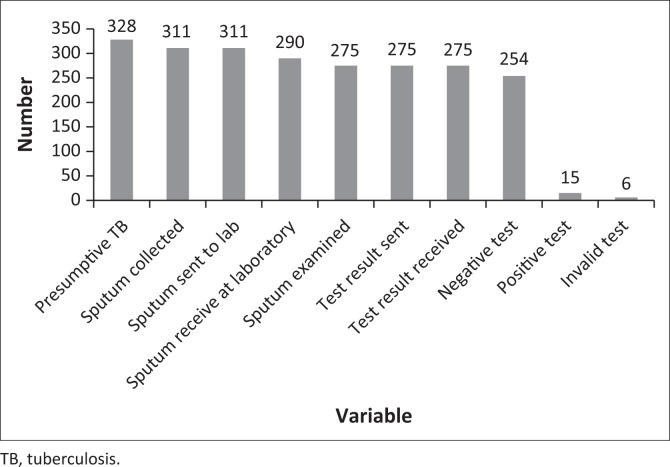
Bar chart of the cascade from presumptive pulmonary tuberculosis patient to receipt of sputum examination results at the non-diagnostic facility in Mpongwe District, Zambia (January–June 2019).

Of the 328 patients with presumptive pulmonary TB, 311 (94.8%, 95% CI 91.8% – 97.0%) submitted sputum at non-diagnostic facilities, representing a loss of 17 (5.2%) at submission stage. All 311 (100%, 95% CI 98.7% – 100%) of the submitted sputum specimens were referred to the diagnostic site for examination. Of the 311 sputum samples that were referred for examination, 290 (93.2%, 95% CI 89.9% – 95.8%) were received at the laboratory, representing a loss of 21 samples (6.8%). Of the 290 samples that were received, 275 (94.8%, 95% CI 91.6% – 97.1%) were examined in the laboratory. The other 15 (5.2%) were rejected for various reasons, including mismatch between names recorded on specimen containers and those recorded on laboratory forms, unlabelled specimen containers, or insufficient sample. Results of all 275 (100%, 95% CI 98.7% – 100%) samples examined in the laboratory were sent back to referring facilities, and all of them (100%, 95% CI 98.7% – 100%) were received. This represents a referral cycle completion rate of 88.4% (95% CI 84.3% – 91.8%). Of the results received 254 (92.4%, 95% CI 88.6% – 95.0%) were negative, 15 (5.5%, 95% CI 3.3% – 8.9%) were positive, and 6 (2.2%, 95% CI 1.0% – 4.8%) were invalid (Figure 1).

Referral cycle completion rate was significantly lower for sputum samples from health facilities located within a 50 km radius from the testing laboratory (84.4%) compared with 94.4% for health facilities outside 50 km radius (*p* = 0.005). There was no significant difference in referral cascade completion rates by availability of staff trained in sputum courier system, courier focal point person, and refrigerator at the referring facility (*p* > 0.05) (Table 3).

**TABLE 3:** Association between sputum specimen referral outcome and attributes of referring health facility in Mpongwe District (January–June 2019), *N* = 328.

Health facility attributes	Referral outcome				Total		*p*
	Completed		Not completed				
	*n*	%	*n*	%	*n*	%	
**Distance to diagnostic facility**							
Below 50 km	157	78.5	43	21.5	200	100	0.001†
50 km and above	118	92.2	10	7.8	128	100	
**Referred from facility with trained staff**							
Yes	134	81.7	30	18.3	164	100	0.294
No	141	86.0	23	14.0	164	100	
**Referred from facility with TB focal point person**							
Yes	154	85.1	27	14.9	181	100	0.498
No	121	82.3	26	17.7	147	100	
**Referred from facility with a refrigerator for sputum storage**							
Yes	129	85.4	31	17.5	151	100	0.470
No	146	82.5	22	14.6	177	100	

TB, tuberculosis.

† , Significant at 0.05 level of significance.

### Turnaround time for sputum samples referred from six non-diagnostic sites

The median TAT (i.e. from sample submission by presumptive TB patient to receipt of results by referring facility) was 8 days (IQR = 3–12 days; range = 0–72 days). The shortest time taken was between sample receipt and examination in laboratory (median TAT = 0 days; IQR = 0–0; range = 0–6 days). The longest time taken was between release of results by the testing laboratory and their receipt at the referring facility (median TAT = 6 days; IQR = 1–8; range = 0–71 days) (Table 4).

**TABLE 4:** Turnaround time for sputum samples referred from six non-diagnostic sites in Mpongwe District (January–June 2019).

Stage in sample referral cascade	Median TAT (IQR) in days	Range (in days)
Between sample submission by presumptive TB patient and referral to diagnostic centre (*n* = 311)	0† (0–0)	0†–10
Between sample referral and receipt at testing laboratory (*n* = 290)	1 (0–2)	0†–15
Between sample receipt and examination in laboratory (*n* = 275)	0† (0–0)	0†–6
Between sample examination and dispatch of results (*n* = 275)	0† (0–0)	0†–3
Between dispatch of results and receipt of results by referring facility (*n* = 275)	6 (1–8)	0†–71
Between sample submission by presumptive TB patient and receipt of results by referring facility (*n* = 275)	8 (3–12)	0†–72

† , Same day action and/or delivery; TAT, turnaround time; TB, tuberculosis; IQR, interquartile range.

### Availability of systems and tools at non-diagnostic facilities

Of the six facilities, four (66.7%) had a focal point person tasked to manage sputum specimen referral system, while two (33.3%) did not. Half (50%) of the facilities did not have staff trained or oriented in the sputum specimen courier system. One-third (33.3%) of the facilities did not have a schedule for sputum specimen pick-up, and none of the facilities had SOPs on sputum collection, packaging and transportation. Five of the six health facilities (88.3%) had sputum containers and absorbent materials (cotton wool or tissue). None of the health facilities had plastic zip bags for the packaging of the sputum samples. Four (66.7%) had no refrigerators for storage of samples. None of the six health facilities had evidence of monitoring and evaluation systems such as reports of meeting minutes on performance of the sputum sample courier system (Table 5).

**TABLE 5:** Availability of systems and tools for operating sputum courier system at six non-diagnostic sites in Mpongwe District (September 2019).

Requirement	Health facility						Availability (*N* = 6)	Percentage
	Kalweo	Ipumbu	Kasamba	Mikata	Machiya	Kanyeda		
Focal person	P	P	X	X	P	P	4	66.7
Trained staff	X	X	P	P	P	X	3	50.0
Pick-up schedule	X	P	P	X	X	X	2	33.3
SOPs	X	X	X	X	X	X	0	0.0
Sputum containers	P	P	P	P	P	X	5	88.3
Absorbent material	P	P	P	P	P	X	5	88.3
Plastic Ziploc bags	X	X	X	X	X	X	0	0.0
Refrigerator	P	X	X	X	X	P	2	33.3
Evaluation evidence	X	X	X	X	X	X	0	0.0
Laboratory forms	P	P	P	P	P	P	6	100.0
Cooler box	X	X	X	X	X	P	1	16.7

SOPs, standard operating procedures; P, available; X, not available.

## Discussion

This study shows that up to 11.6% of the sputum samples collected from presumptive pulmonary TB patients in Mpongwe District between January and June 2019 were lost along the sputum referral cascade. This figure was lower than what was found in similar studies in Malawi, Tanzania and Zimbabwe where 60%, 91% and 28% of sputum samples, respectively, got lost along the referral cascade.^5,13,14^ Our results were however higher than the 7% reported by another study conducted in the Southern Province of Zambia.^8^ The loss of sputum samples along the referral cascade has key programmatic and public health implications as it leads to delays in diagnosing and initiating treatment in patients with TB.^3^ This in turn could perpetuate further transmission of TB, including the drug-resistant TB, which is a major public health concern. Evidence suggests that a person with infectious TB can infect an estimated 10–15 other people in a year.^5^ Mpongwe District Health Office must therefore make concerted efforts to strengthen the sputum sample referral system.

Studies have found long distances to testing laboratories to be one of the factors that contribute to loss of sputum samples along the referral cascade.^5,6,8^ To the contrary, this study found that referral cascade completion rate was significantly higher for sputum specimens referred from health facilities located within 50 km from the testing laboratory compared with those located outside 50 km. Factors such as service providers’ attitudes and practices, which were not investigated by this study, could have contributed to this finding.

The largest loss (6.8%) in this study occurred between dispatch of sputum samples from non-diagnostic centres and receipt at the testing laboratory. This finding was consistent with results from Zimbabwe.^5^ The reasons for loss of sputum samples at this stage are not very clear, but studies conducted in other settings have attributed this to weaknesses in the sputum transportation system and shortcomings in recording.^3,14^ Further operational research may be needed to establish how sputum samples are lost at this stage of the referral cascade. Introduction of quarterly monitoring and reporting of gaps in sputum samples referral cascade and regular supervisory visits to non-diagnostic sites and the testing laboratory may also help to identify areas that need improvement.

The second largest loss along the sputum sample referral cascade for Mpongwe occurred at sputum specimen submission stage. Up to 5.2% presumptive pulmonary TB patients did not provide sputum. This could in part be attributed to patients being unable to cough out sputum. Educating patients on coughing techniques and counselling them on the importance of providing sputum may help to reduce losses at this stage of the cascade. Studies have shown that integrating counselling into TB care generates better patient adherence to treatment protocols.^15^ But the lack of sputum packaging materials such as Ziploc bags and refrigerators in the study sites may also discourage health workers from collecting sputum samples from patients.

Our study also found that 5.2% of the samples received at the laboratory were rejected (not tested). Common reasons for sample rejection included mismatch between names recorded on specimen containers and those recorded on laboratory forms, unlabelled specimen containers and insufficient sample. All these factors may be an indication of inadequate knowledge in sputum collection, packaging and transportation among staff in non-diagnostic centres. Orientation of staff in sputum collection, packaging and transportation and provision of SOPs may help to reduce sputum sample rejection rates. In Uganda, for example, training of staff in specimen courier system coupled with provision of SOPs, packaging materials and transport helped increase the proportion of sputum samples completing the referral cascade.^3^

Studies from other settings have also found losses of sputum specimen results after they have been released from the testing laboratory.^5,8^ While it was encouraging that the results from this study showed all the sputum samples tested in the laboratory were received at the referring facility, this finding was at the same time concerning because transportation of both sputum samples (some of which were lost along the way) and results (all of which were successfully delivered) were performed using the same motorbike courier system. Further operational research is required to investigate this discrepancy.

Another encouraging finding from this study was the sputum specimen TAT, which averaged (median) six days and was within the nine day timeframe recommended by the Global Laboratory Initiative.^6^ Furthermore, the TAT in this study was shorter than the seven days that was reported in Zimbabwe.^5^ Shorter TATs may lead to quicker initiation of treatment for patients with TB, reduce mortality associated with late TB treatment initiation and reduce community transmission.

Notwithstanding the shorter TAT found by this study, the delay between obtaining a result and receipt by the referring centre seems surprisingly and unnecessarily long as the information could be transmitted via phone call while waiting for the courier to deliver paper-based results. Staff should take advantage of mobile phone connectivity in their areas to follow-up of results via phone calls. Results received via phone call should be taken as official, documented in patient registers, and later on verified using paper forms delivered by the courier.

A bonus programme quality finding by this study was that 22% of presumptive TB patients were of unknown HIV status, against the national TB programme guidelines that require all presumptive TB patients to be offered an HIV test.^16^ The 2012 WHO policy on TB and HIV collaborative activities also recommends for routine provider-initiated HIV testing and counselling (PITC) to both diagnosed TB patients and persons with presumptive TB in order to promote early identification and treatment of HIV to reduce the HIV burden in TB patients.^17^

In Zimbabwe, high level of HIV ascertainment rate was attained by: (1) implementation of the integrated TB-HIV services wherein presumptive TB patients are offered both TB and HIV testing simultaneously, (2) deployment of full-time primary care counsellors who provide HIV counselling and testing services at all high volume health facilities, (3) decentralized availability of rapid HIV test kits in all public health facilities, at no cost to the patient.^18^ Mpongwe District Health Office should consider implementing or scaling up such initiatives.

To the best of our knowledge, this is the third study in Zambia to track the movement of referred sputum samples. The study provides useful information and a framework that can be used to evaluate sputum courier systems in other settings. Because of the small number of health facilities involved, coupled with the use of non-probability sampling, results from this study may not be generalisable to other settings. This is one of the major limitations of our study. The other limitation was the missing dates in registers at almost all the stages of the referral cascade. This reduced the number of samples included in the calculation of TATs. Additionally, we did not investigate attitudes and practices of healthcare workers, which may also contribute to loss of sputum specimens along the referral cascade. Broader research is recommended to understand how factors not investigated by this study may contribute to loss of sputum samples along the referral cascade.

## Conclusion

This study has shown that, in general, the sputum sample referral system for Mpongwe District was performing well. Notwithstanding this result, some samples were getting lost at three points: (1) sputum submission, (2) sputum transportation and (3) sputum testing in the laboratory. Inadequate staff trained in sputum collection and packaging and weaknesses in transportation and monitoring systems could be some of the factors contributing to losses in the referral cascade for Mpongwe District. Other factors include the lack of SOPs on sputum handling and lack of packaging materials such as Ziploc bags. Mpongwe District Health Office should put in place measures to avoid losses in the sputum sample referral cascade, including developing a strong system (with SOPs) to regularly monitor and evaluate the movement of sputum samples along the referral cascade. Staff in non-diagnostic facilities should also be trained in sputum sample collection and regularly supervised to ensure accuracy in recording and reporting. The District Health Office should also consider carrying out a follow-up study that will involve all health facilities in the district, including those without a formalised courier system. This will give a holistic view of performance of the referral system, and increase generalisability of the findings.
